# Ecological integrity as a foundation for European health policy

**DOI:** 10.1016/j.lanepe.2026.101833

**Published:** 2026-08-24

**Authors:** Nikola Biller-Andorno, Migle Laukyte, Barbara Prainsack

**Affiliations:** a Institute of Biomedical Ethics and History of Medicine, University of Zurich, Winterthurerstrasse 30, 8006, Zurich, Switzerland; b Department of Law, Pompeu Fabra University, Ramon Trias Fargas 25–27, 08005, Barcelona, Spain; c Department of Political Science, University of Vienna, Universitätsstrasse 7, 1010, Vienna, Austria

Twenty years after the Council of the European Union endorsed overarching values for health systems, Frischhut and colleagues have proposed updating them in response to demographic, technological, geopolitical, and environmental change.^1^ Their proposals rightly enlarge the ethical frame of European health policy. Yet one foundation remains insufficiently articulated: the integrity of the ecological systems on which health depends. The European Group on Ethics in Science and New Technologies (EGE), the European Commission's independent ethics advisory body, called for a right to a clean, healthy, and sustainable environment and for plural ways of valuing nature.^2^ We argue that ecological integrity supplies a necessary normative and material foundation for making that right consequential in health policy.

We use ecological integrity to denote the capacity of ecological systems to sustain biodiversity, essential functions, adaptive resilience, and regenerative processes over time.^3^ In ecological science, ecosystem integrity is used similarly. Svenning's dynamic account sharpens it by emphasising the continuation of biodiversity-generating processes and evolutionary lineages through transformation.^4^ Integrity therefore does not imply pristine nature, an unchanging species composition, or the exclusion of responsible human use.

Why not rely on familiar terms? Sustainability is an important but broad aim and can permit ecological losses to be balanced against, or substituted by, economic and social gains. Resilience cannot provide the missing normative anchor. It describes one property of a system, and a severely degraded state can itself be resilient. Plural valuation shows why nature matters in different ways, including relational, intrinsic, and instrumental values.^5^ Ecological integrity specifies what must endure and when prospective loss should constrain choice.

Ecological integrity has three connected roles. It is a normative commitment because ecological systems matter beyond the benefits they provide to humans. It is a biophysical condition of present and future health because functioning living systems regulate climate, water, soils, food production, and disease. It is also a constraint on decision making. Where ecological boundaries or risks of serious or irreversible damage are engaged, projected health or economic gains cannot be assumed to compensate for ecological loss and should not automatically override those constraints.

This idea is not foreign to EU governance. The EGE's fifth recommendation states that economic valuation should operate within ethical and ecological conditions so that it supports “wellbeing, resilience, ecological integrity, and multiple ways of valuing nature”.^2^ The Nature Restoration Regulation likewise uses ecological integrity in defining the good condition of habitats.^6^ The term therefore appears in both independent EU ethics advice and binding EU law. Our proposal extends this existing vocabulary by treating ecological integrity as a foundation for health policy, rather than only as an environmental objective or a consideration within valuation.

The UN General Assembly recognised the right to a clean, healthy, and sustainable environment in 2022.^7^ It complements the right to health, which already encompasses environmental determinants of health,^8^ but is not interchangeable with it. The right to a healthy environment makes environmental quality itself an object of entitlement and can encompass ecological and intergenerational interests not fully captured by health metrics. Ecological integrity gives substantive content to what a healthy environment requires; the right can translate that foundation into duties, participation, accountability, and remedy.^9^

The Commission's Better Regulation framework offers an immediate route to implementation.^10^ Impact assessments should identify effects on ecological diversity, functions, resilience, and regenerative capacity; establish whether ecological boundaries or risks of serious or irreversible harm are engaged; and explain whose knowledge and values informed the appraisal. Health effects and monetary valuation remain necessary, but should operate within those constraints rather than decide whether the constraints apply.

Ecological integrity is therefore not simply one more value alongside dignity, equity, solidarity, or access to care. It sets conditions for their durable realisation and has priority when a policy would cross ecological boundaries or create a serious risk of irreversible harm. European health policy cannot credibly protect health today by compromising the living systems on which health tomorrow depends. Panel 1 devised by the authors.

Panel 1 **What ecological integrity adds to health-policy decisions**

**Object of protection:** The diversity, functions, resilience, and regenerative capacity of ecological systems, not only their measurable benefits for human health.

**Decision rule:** Ecological boundaries and serious risks of irreversible harm delimit permissible options rather than entering appraisal only as costs that can always be offset.

**Institutional test:** EU impact assessments should document ecological effects, plural valuation and representation, the justification for trade-offs, and arrangements for review and remedy.

## Contributors

NBA conceived the Comment and prepared the original draft. NBA, ML, and BP contributed to the conceptual development of the argument, the interpretation of the relevant ethical, legal, scientific, and policy literature, and revision of the manuscript. NBA developed the initial conceptual panel, which all authors reviewed and revised. All authors reviewed and approved the final manuscript and accept responsibility for its content.

This Comment reports no original data. The authors reviewed and verified the sources relevant to their contributions.

## Declaration of generative AI and AI-assisted technologies in the writing process

During preparation of this Comment, the authors used OpenAI's ChatGPT to support language editing, manuscript organisation, and critical review. The authors independently verified the sources and claims, reviewed and edited the manuscript, and take full responsibility for its content.

## Declaration of interests

BP is Chair, and NBA and ML are members, of the European Group on Ethics in Science and New Technologies (EGE); all three contributed to the EGE Opinion Valuing Nature: Implications for EU Governance. BP is a coauthor of the article cited as reference 1. We declare no other competing interests.
